# Reward Sensitivity Modulates Brain Activity in the Prefrontal Cortex, ACC and Striatum during Task Switching

**DOI:** 10.1371/journal.pone.0123073

**Published:** 2015-04-13

**Authors:** Paola Fuentes-Claramonte, César Ávila, Aina Rodríguez-Pujadas, Noelia Ventura-Campos, Juan C. Bustamante, Víctor Costumero, Patricia Rosell-Negre, Alfonso Barrós-Loscertales

**Affiliations:** 1 Departament de Psicologia Bàsica, Clínica i Psicobiologia, Universitat Jaume I, Castelló de la Plana, Spain; 2 Departamento de Psicología y Sociología, Facultad de Educación, Universidad de Zaragoza, Zaragoza, Spain; University of Western Ontario, CANADA

## Abstract

Current perspectives on cognitive control acknowledge that individual differences in motivational dispositions may modulate cognitive processes in the absence of reward contingencies. This work aimed to study the relationship between individual differences in Behavioral Activation System (BAS) sensitivity and the neural underpinnings involved in processing a switching cue in a task-switching paradigm. BAS sensitivity was hypothesized to modulate brain activity in frontal regions, ACC and the striatum. Twenty-eight healthy participants underwent fMRI while performing a switching task, which elicited activity in fronto-striatal regions during the processing of the switch cue. BAS sensitivity was negatively associated with activity in the lateral prefrontal cortex, anterior cingulate cortex and the ventral striatum. Combined with previous results, our data indicate that BAS sensitivity modulates the neurocognitive processes involved in task switching in a complex manner depending on task demands. Therefore, individual differences in motivational dispositions may influence cognitive processing in the absence of reward contingencies.

## Introduction

Cognitive control can be defined as “the ability to regulate, coordinate and sequence thoughts and actions in accordance with internally maintained behavioral goals” [1]. Current approaches in the study of the interaction between cognitive and motivational processes highlight the importance of taking into account individual differences in affective or motivational factors [1,2]. Two different approaches have been used to investigate this interaction. One consists in comparing the executive tasks performed under rewarded and non-rewarded conditions [3]. The other, which is the strategy employed in this study, investigates how individual differences in reward sensitivity modulate performance in executive tasks.

A recent review has identified the most relevant brain structures involved in the interaction between cognition and motivation, which comprise the striatum, the anterior cingulate cortex (ACC) and the lateral prefrontal cortex [4]. The striatum has been proposed to support a gating mechanism that updates goal representations in the prefrontal cortex [5]. This role is mediated by dopaminergic neurotransmission in that increases in striatal dopamine favor the flexible updating of current task-relevant representations, which results in enhanced cognitive flexibility [6]. The role of the ACC is to evaluate the relative costs and benefits of engaging in effortful, controlled rather than automatic processing [7]. Finally in non-rewarded contexts, lateral PFC activity has been found to reflect the subjective cost associated with exerting cognitive control [4]. So, the role of the lateral PFC is more prominent when control demands of the task are higher. This effect has been proposed to be complexly mediated by dopaminergic neurotransmission [5]. When control demands are high, the increment in dopamine levels in the striatum favors cognitive flexibility, while dopaminergic input in the lateral prefrontal cortex favors cognitive focusing [5,6].

Different personality models, such as those proposed by Gray [8], Depue and Collins [9], Cloninger [10] and Fowles [11], describe a “Behavioral Activation System” (BAS) as a motivational system that underlies individual differences in reward sensitivity, incentive motivation and impulsivity [8,12,13], and it has dopaminergic neurotransmission as its proposed biological basis [8,14]. Individuals with a highly active BAS tend to show more positive affect, are more sensitive to, and more likely to approach, reward, even in the presence of aversive or irrelevant stimuli, than those with a less active BAS [15]. Consequently, individuals with stronger reward sensitivity are more aroused in the presence of cues of reward, tend to more intensely focus on rewarded and goal-directed behaviors and show higher striatal activity in the presence of reward cues [16–18]. However, the influence of BAS on behavior is more complex than its effects on motivational reactivity, and further theoretical developments have moved on to highlight the role that BAS plays in executive functions, such as working memory and task switching, in the absence of reward contingencies [19,20].

What is the relationship between the BAS and cognitive function in the absence of reward contingencies like? According to Aarts et al. [6], appetitive motivation appears to have parallel effects to those of increases in striatal dopamine, that is, the enhancement of cognitive flexibility, which may come, however, at the expense of reduced cognitive focusing (i.e., greater distractibility). This view is consistent with previous behavioral studies which have shown enhanced cognitive flexibility in terms of reduced switch costs [21] and decreased distractor processing [22] in individuals with higher BAS sensitivity during tasks that require high, fast control demands. However, the association between the BAS and cognitive control seems to be more complex. While individuals with stronger reward sensitivity have shown enhanced cognitive flexibility during tasks that require high control demands (see above), some behavioral studies have also shown better conscious cognitive focusing in other tasks that involve lower control demands [23,24]. A BAS-related trait such as Extraversion has been associated with better task performance in a n-back task, but only under dual task conditions and higher working memory load [25].

In neural terms, a previous study has shown that reward sensitivity is associated with increased activity in the striatum and the right inferior frontal cortex, as well as reduced activity in the ACC during a difficult switch task [19]. It is noteworthy that these differences were found in a context with a high proportion of switch trials (50%) and simultaneous presentation of task-cues and target stimuli, so participants had to quickly and frequently update the response set. Similarly, a previous study has found that sustained activity of the ACC and the lateral prefrontal cortex during the n-back task correlated negatively with BAS scores [20]. In accordance with the classical arousal theory, which explains that extraverts outperform introverts during difficult tasks, the authors proposed a neural efficiency theory that linked the BAS to lower neural activation in target brain areas (i.e., ACC and lateral prefrontal cortex) during cognitive tasks that required sustained control. Thus, while the findings for the ACC seem to be quite consistent, there is a discrepancy regarding the lateral prefrontal cortex. Given that this region seems to be sensitive to the costs of engaging in controlled processing [4], the different association of the BAS and lateral prefrontal cortex in these previous studies might be linked with the different demands of each task. Therefore, the influence of the BAS on behavior and brain activity during cognitive control might be dependent on task demands [6].

The present study was designed to complement our previous results [19]. To this end, we adapted a different task-switching paradigm with a low proportion of switches from Barceló, Periáñez, and Nyhus [26]. We used an intermittently-instructed switching paradigm [27] in which a cue was randomly presented before each series of trials to inform participants if the response rules would be the same as, or would differ from, the preceding series. This design had two advantages: first, switch and repeat cues were presented after a series of trials, which allowed the stabilization of the current task set to thus result in low-frequency task-switching. Previous studies have suggested that this type of paradigm may allow a better observation of the brain activity associated with the switch cue compared with those tasks in which the attentional set has to be frequently changed (50% of trials) [28–31]. Second, the switch and repeat cues were presented before the actual target stimulus and required no motor responses. Therefore, we were able to study the processing of the switch cue and the updating of the attentional set without the potential confounds that involve target encoding and motor responses. Previous behavioral and psychophysiological studies have associated the processing of the switch cue with proactive control and set-switching processes to form a representation of stimulus-response associations in working memory [32–34]. Hence in this work, our aim was to study if individual differences in BAS sensitivity modulate brain activity during the processing of the switch cue in a switching task requiring lower frequency of switches.

Given the above-mentioned antecedents, we have put forward our hypothesis for the task and BAS correlates. According to the proposed role of the striatum in cognitive flexibility, processing the switching cue should elicit striatal activity. Moreover, since the updating of task rules may also involve working memory and task monitoring, we also expect to find lateral prefrontal and ACC activity. Finally, individual differences in the BAS are expected to modulate the activity of these regions. If greater BAS sensitivity implies enhanced cognitive flexibility, we may observe increased striatal activity in high-BAS individuals. On the other hand, we expect to find a negative association between the BAS and lateral prefrontal cortex and ACC activity, which is consistent with previous accounts of reduced activity in those regions that have associated the BAS with greater neural efficiency [20,35].

## Materials and Methods

### Ethics statement

Participants provided written informed consent prior to participation in the experiment, and the study was approved by the Universitat Jaume I Ethical Committee.

### Participants

Twenty-eight right-handed, healthy undergraduate students (15 females; *M*_age_ = 24.21, *SD* = 4.08; range: 19–32) participated in this study. All participants had normal or corrected-to-normal vision, and no history of previous or current neurological disease or head trauma with loss of consciousness. None of the participants had an Axis I or Axis II diagnosis. Each participant received a monetary reward for his or her participation (€30).

### BAS Assessment

Following the methodology of our previous work [19], participants completed the Sensitivity to Reward (SR) scale from the Sensitivity to Punishment and Sensitivity to Reward Questionnaire (SPSRQ; [36]) as a measure of BAS sensitivity. This scale was chosen for the good psychometric properties that it has shown in previous studies [37,38]. The SR scale mean score was 9.86 (*SD* = 3.95), which is in accordance with previous reports [36,37]. The scale was normally distributed according to the Shapiro-Wilk test and showed good reliability as measured by Cronbach’s alpha (α = .73). As the interest of this study lay in the role of individual differences in the BAS on brain activation during task-switching, only the SR measures were considered in our analysis.

### Task

A nonlinguistic switching task devised by Barceló et al. [26] was adapted for the fMRI scanner using a slow event-related design based on an intermittently-instructed task-cuing paradigm (see [27] for a description of different task-switching paradigms). Visual stimuli consisted of four equally probable colored shapes (red and blue circles and squares; *p* = .225 each) and two infrequent black shapes (vertical dollar sign and horizontal dollar sign; *p* = .05 each). The interstimulus interval was set at 1,500 ms, and each stimulus lasted 500 ms. Colored shapes acted as response stimuli, whereas black shapes acted as switch or repeat cues.

**Fig 1:**
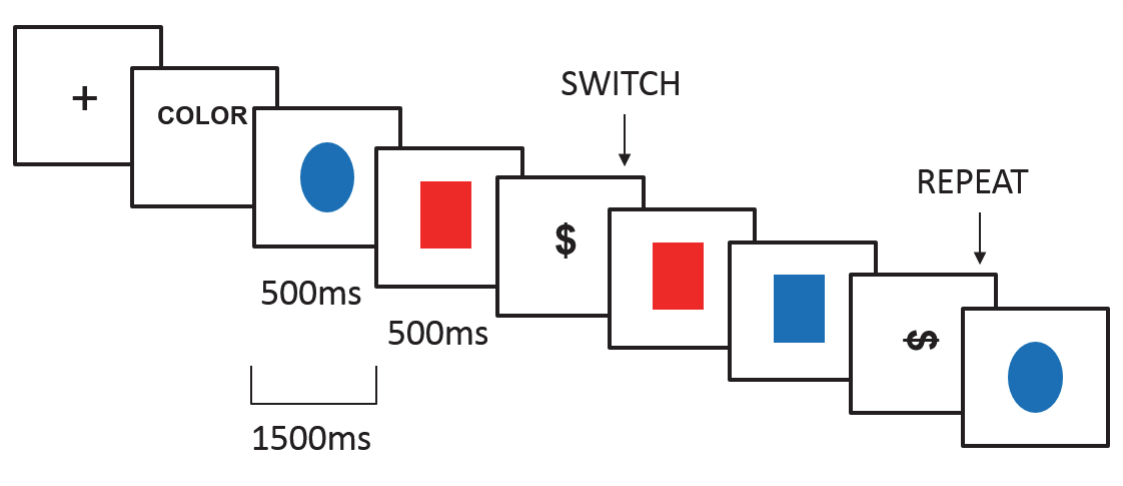
Switching task. Each run started with a fixation cross followed by a written label indicating the response rule: *color* or *forma* (*shape* in Spanish). Subsequent trials started with a switch cue (vertical dollar sign), which indicated a change in the response rule, or a repeat cue (horizontal dollar sign), which indicated repetition of the response rule. The cue was followed by a variable number (8–12) of response stimuli (blue and red circles and squares). The cue always referred to the response rule of the previous trial, independently of the instructions presented at the beginning of the run.

A trial was defined as a cue and a sequence of response stimuli until the next cue appeared (Fig 1). To make the presentation of the events of interest (cues) unpredictable, each trial had a varying number of response stimuli (8, 9, 10, 11 or 12) with a mean duration of 14.5 s (*SD* = 4.04). During the scanning session, each participant performed 70 trials distributed into five runs (each consisting of 14 trials lasting 4:05 min).

The participant’s task was to sort the four colored shapes according to two classification rules (either color or shape). At the beginning of each run, a written label was presented to inform the participant about the initial response rule. The label *color* informed participants that they had to respond according to the color of the response stimulus; the label *forma* (*shape* in Spanish) informed participants that they had to respond according to the shape of the response stimulus. Depending on the sorting rule, participants responded by pressing a button with their right index finger (red color, circular shape) or right thumb (blue color, square shape) for a total of 370 responses with each finger among the five runs. The appearance of a black cue indicated the start of a new trial. A vertical dollar sign acted as the *switch* cue, indicating a change in the response rule with respect to the previous trial (e.g., if the previous response rule was color, the participant had to respond according to shape after seeing the vertical dollar sign). A horizontal dollar sign was a *repeat* cue, indicating that the previous response rule had to be maintained in the subsequent trial (see Fig 1). The cues always made reference to the response rule used in the previous trial, independently of the explicit label presented at the beginning of the run. The speed and accuracy of each behavioral response were registered. Before the scanning session, participants completed a 5-min practice session to ensure they understood the instructions.

In order to avoid inaccurate scoring of task-switching errors at rule transition points (after a black symbol), the response stimuli before and after cues were always a red square or a blue circle. This allowed for unambiguous assignment of motor responses with correct or incorrect classification rules since the button response was unique to the rule. The manual response was counterbalanced for the first and second responses after the cue, and for the response before the cue. To keep perceptual priming effects constant across conditions, we controlled for the sequential probabilities between each pair of stimuli. The global probability of two successive repeat cues was the same as that of two successive switch cues. Likewise, the global probability of a switch cue being followed by a repeat cue equaled the probability of a repeat cue being followed by a switch cue. Finally, there was the same number of trials (35 of each in the five runs) for both cues.

The task was programmed and presented using Presentation software (Neurobehavioral Systems, Inc., Albany, CA) running in a Microsoft Windows XP operating system. Visual stimuli were displayed inside the scanner using Visuastim goggles (Resonance Technology, Inc., Northridge, CA), stimuli presentation was synchronized with the scanner using a SyncBox (Nordic NeuroLab AS, Bergen, Norway) and responses were registered with a ResponseGrip (Nordic Neurolab AS, Bergen, Norway) recording device.

### Behavioral Measures during fMRI Task Performance

The percentage of errors was calculated for each task condition and reaction times (RTs) were estimated only from correct trials. The switch cost was computed as the difference in mean RTs between the first stimuli after the switch and repeat cues. Only the first stimulus after the cue was included in the analyses because first target trials present maximal effects of switch-specific local costs [26,27]. Mean RTs and error rates were subjected to a paired *t* test to analyze the existence of significant differences between switch and repeat trials. Moreover, we conducted correlation analyses between SR scores and switch costs, error rates and RTs.

### FMRI Acquisition

All experimental sessions were performed in a 1.5 T scanner (Siemens Avanto, Erlangen, Germany). Participants lay in the scanner in a supine position and their heads were immobilized with cushions. A BOLD echo-planar imaging (EPI) sequence of 98 volumes per run was used for fMRI (TE = 50 ms, TR = 2500 ms, FOV = 224 × 224 mm, matrix = 64 × 64, voxel size = 3.5 × 3.5 × 4, slice thickness = 3.5 mm, slice gap = 0.5 mm, flip angle = 90º). We acquired 28 interleaved axial slices parallel to the anterior-posterior commissure plane covering the entire brain. Prior to the functional magnetic resonance sequence, a T1-weighted anatomical 3D gradient-echo pulse sequence was acquired (TE = 4.9 ms, TR = 11 ms, FOV = 24 cm, matrix = 256 × 224 × 176, voxel size 1 × 1 × 1, slice thickness = 1 mm).

### Image Analysis

Image processing and statistical analyses were carried out using SPM5 (Wellcome Trust Centre for Neuroimaging, London, UK). Each participant’s images were first temporally aligned across the brain volume by slice-timing correction. Then, images were realigned and resliced onto the mean EPI image to correct for head motion. Afterward, the corresponding anatomical (T1-weighted) image was coregistered to the mean EPI image. The functional volumes were normalized (voxels rescaled to 3 mm^3^) with the normalization parameters obtained after anatomical segmentation within a standard stereotactic space (the T1-weighted template from the Montreal Neurological Institute). Finally, functional volumes were smoothed using an 8-mm FWHM Gaussian kernel.

Image analyses were performed using a general linear model. In the first-level analysis, the responses to the switch cue and the repeat cue were modeled separately after convolving each event-related unit impulse with a canonical hemodynamic response function and its temporal derivative. Each kind of response stimulus (red circle, blue circle, red square, blue square) was also modeled separately. Realignment parameters were included for each participant as regressors of noninterest. A high-pass filter (128 s) was applied to the functional data to eliminate low-frequency components. Contrast images of the parameter estimates were computed to directly compare both cues (switch cue > repeat cue).

In the second-level random effects analysis, the resulting contrast images of the parameter estimates were used to test the functional main effects of the task and their association with individual differences in reward sensitivity. First, a one-sample *t*-test was run to see which brain regions were more active during the processing of the switch cue compared with the repeat cue. Second, a regression analysis was conducted with SR scores as the regressor of interest. The results are reported with a statistical threshold of *p* < .05, FWE-cluster corrected (voxel-level uncorrected threshold of *p* < .001, minimum cluster size = 30 voxels). Given that reward sensitivity has been previously shown to modulate the BOLD response in the ventral striatum [18], we built an ROI that corresponded to this area as a 6-mm-radius sphere centered at +/-10, 8, -4 (*x*, *y*, *z*, MNI coordinates based on [18], [39], and [40]). Then we extracted the first eigenvariate from the one-sample *t*-test for this region and entered it in the correlation analyses with the SR scores.

We also conducted correlation analyses between the brain regions identified in the switch cue > repeat cue contrast and switch costs (in terms of switch vs. non-switch RT and error rates) to explore the association between brain reactivity to the switch cues and the behavioral switch cost associated with the target stimulus. Brain activation was extracted by building 6mm spheres around the task local maxima from which the first eigenvariate for the one-sample t-test was extracted.

## Results

### Behavioral Performance

Mean RTs, switch costs and error rates are summarized in Table 1. The switch cost analysis revealed that the RT to the first response stimulus after the cue was longer for switch trials than for repeat trials, *t*_(27)_ = 5.99, *p* < .001. The switch trials also yielded a higher error rate than the repeat trials, *t*_(27)_ = 4.05, *p* < .001. The correlations between SR scores and switch costs, RTs and error rates (globally and for each condition independently) were all nonsignificant (*p* >. 1).

**Table 1:** Means and Standard Deviations of RTs, Switch Costs and Error Rates.

	*****M*****(*****SD*****)****	****Correlation with SR Scores (Pearson’s*****r*****)**** ^a^
**RTs (in ms)**		
Overall task	400 (49)	.01
Switch trials	483 (103)	.02
Repeat trials	434 (73)	.01
**Error rates (in %)**		
Overall task	9.79 (5.39)	.10
Switch trials	17.76 (11.67)	-.15
Repeat trials	11.12 (9.59)	.04
**Switch costs**		
RT (in ms)	49 (43)	.09
Accuracy (in %)	6.63 (8.66)	.19

*Note*. RT = reaction time.

^a^ *p* >. 05.

### Imaging Data

**Fig 2:**
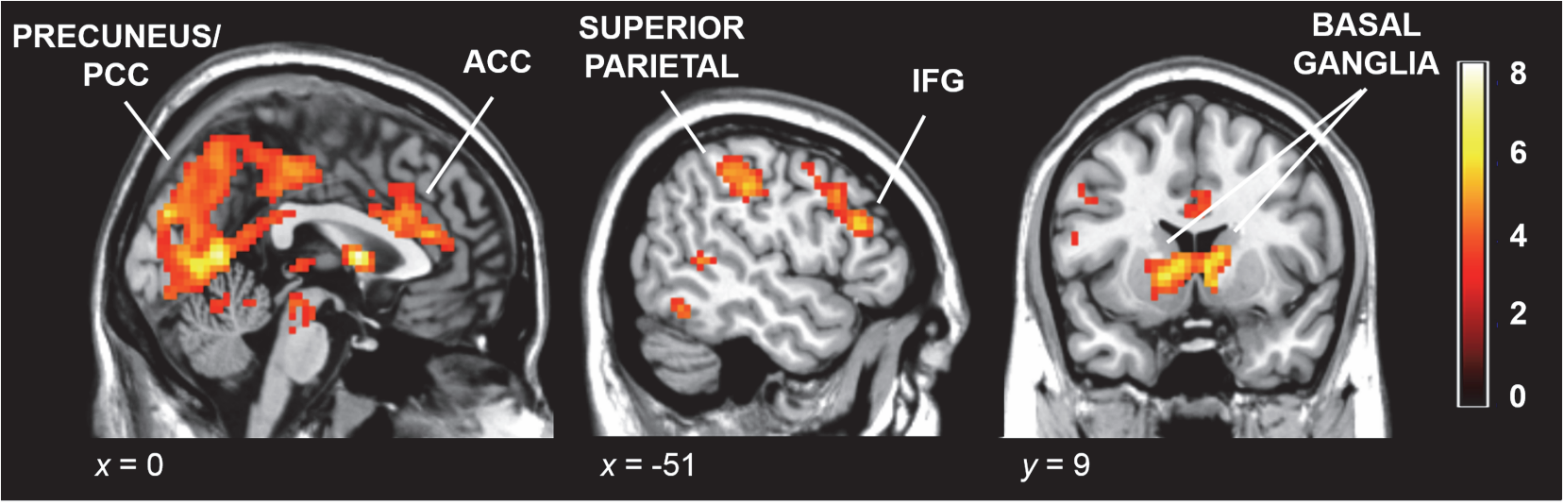
Whole-brain results. Average activation maps for the Switch > Repeat contrast overlaid on a standard brain (*p* < .05, cluster corrected with an auxiliary uncorrected threshold of *p* < .001). The right side of the image is the right side of the brain. The color bar depicts *t* values. PCC = posterior cingulate cortex; ACC = anterior cingulate cortex; IFG = inferior frontal gyrus.

The group-average maps for the task showed greater activation for switch cues than for repeat cues in regions within the frontostriatal network, including the IFG, DLPFC, ACC and bilateral caudate nuclei extending into the left putamen, as well as in parietal regions and occipital visual areas (see Table 2 and Fig 2). The opposite contrast showed no regions with significantly greater activation for the repeat condition than for the switch condition. No region’s activation during the processing of the switch cue was correlated with the behavioral switch cost either in terms of RT or error rate.

**Table 2:** Brain Areas Showing Greater Activity for the Switch Condition than the Repeat Condition.

****Region****	****BA****	****Hemisphere****	****Coordinates (x, y, z)****	*****t*****	****Cluster Size (*****k*****)****
Striatum		B	0 3 9	8.24	604
			9 3 6	7.70	
			-12 9 0	6.83	
Superior parietal cortex	40	L	-48–24 39	6.18	367
			-42–45 33	5.78	
			-48–33 42	5.52	
Inferior frontal gyrus	9/45	L	-54 24 21	6.09	117
Inferior frontal gyrus			-51 15 36	5.08	
Precentral gyrus			-42–6 54	4.78	
DLPFC		R	39 36 21	4.14	40
			33 45 24	4.02	
ACC	24/32	R	0 36 18	5.72	173
			0 21 24	5.24	
			6 15 36	4.26	
Occipital area/Precuneus	30/7/18	B	-3–69 3	8.47	3356
			3–60 9	8.27	
			-9–33 48	7.74	
Occipital area	19	R	42–81–6	5.37	124
			42–81 6	5.04	
			51–72–9	4.03	

Coordinates are reported in the Montreal Neurological Institute space. The *t* values refer to the three local maxima within each cluster (*p* < .05 cluster-corrected). BA = Brodmann area; B = both hemispheres; L = left hemisphere; DLPFC = dorsolateral prefrontal cortex; R = right hemisphere; ACC = anterior cingulate cortex.

**Fig 3:**
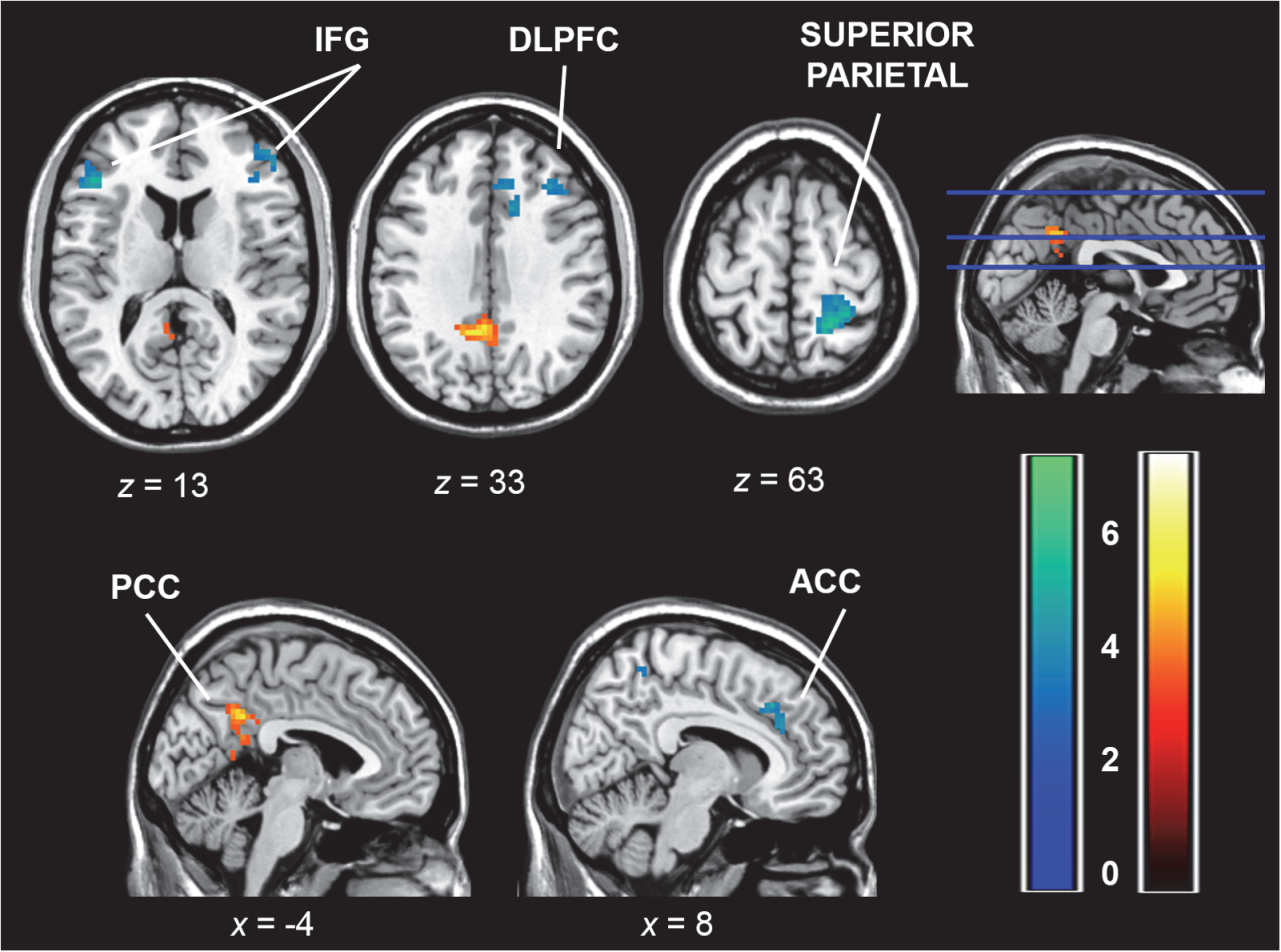
Regression analysis. Whole-brain correlation maps between brain activity in the Switch > Repeat contrast and SR scores (*p* < .05, cluster corrected with an auxiliary threshold of *p* < .001). Color bars depict negative (blue-green) and positive (red-yellow) *t* values. The right side of the image is the right side of the brain. PCC = posterior cingulate cortex; ACC = anterior cingulate cortex; IFG: inferior frontal gyrus; DLPFC: dorsolateral prefrontal cortex.

The regression analysis revealed a positive correlation between SR scores and brain activity in the switch versus repeat condition in the posterior cingulate cortex (PCC). Negative correlations were found between SR scores and the Switch > Repeat contrast for brain activity in the IFG, DLPFC and ACC, as well as in the inferior parietal cortex and postcentral gyrus (Table 3, Fig 3). These results did not change significantly when the switch costs in terms of RT or errors were entered as covariates in the regression analysis, separately. In other words, the correlations between brain activity and SR scores were still significant after controlling for the effect of behavioral differences. Therefore, switching between tasks was associated with a RT and accuracy cost but, likely, the length of the foreperiod allowed an advanced reconfiguration which reduced the effects on task execution [34].

**Table 3:** Brain Areas Showing a Correlation between SR Scores and Activation in the Switch > Repeat Contrast.

**Correlation/Region**	**BA**	**Hemisphere**	**Coordinates (x, y, z)**	***t***	**Cluster Size (*k*)**
**Positive**					
Posterior cingulate cortex	31	L	-9–51 33	5.41	106
			-6–45 15	4.21	
			-3–54 12	3.66	
**Negative**					
Superior parietal cortex	5	R	21–48 66	7.27	77
			30–42 63	5.41	
			18–42 57	4.34	
Inferior frontal gyrus	46	L	-45 30 12	5.45	30
ACC	6/32	R	6 27 39	4.99	53
			15 24 36	4.80	
			9 33 27	4.29	
Inferior frontal gyrus	45	R	48 42 9	4.62	46
			39 27 15	3.89	
DLPFC	46		30 42 24	3.74	34
			33 30 33	4.35	
			42 27 33	3.85	
Supramarginal gyrus	40	R	57–30 36	5.37	90
			48–15 42	4.24	
			48–12 57	3.63	
Postcentral gyrus	40	L	-36–33 45	5.24	54
			-33–24 57	4.33	
			-30–9 51	3.74	

Coordinates are reported in the Montreal Neurological Institute space. The *t* values refer to the three local maxima within each cluster (*p* < .05 corrected at the cluster level). BA = Brodmann area; L = left hemisphere; R = right hemisphere; ACC = anterior cingulate cortex; DLPFC = dorsolateral prefrontal cortex.

The ROI analysis yielded a significant negative correlation between the SR scores and ventral striatal activity while processing the switch cue in the left ventral striatum (*r* = -.54, *p* = 0.003), while the correlation in the right ventral striatum was not significant (*r* = .25, *p* = .19). These results remained unchanged, even when controlling for the effect of switch costs.

## Discussion

This study was designed to investigate the influence of BAS-related individual differences on the brain mechanisms involved in cognitive control. We adapted a switch task for fMRI, which was designed to study control processes after a cue signaling to switch the cognitive set. Presentation of switch cues produced the expected activations in the IFG, DLPFC, ACC, striatum and superior parietal cortex when compared with repeat cues. Crucially, individual differences in reward sensitivity correlated negatively with activity in the ventral striatum, ACC and lateral prefrontal areas involved in task switching.

### Processing of the Switch Cue

The switching task used in this study yielded the expected results. The behavioral analyses showed that the magnitude of switch costs and RTs was similar to that of the original task implementation using an ERP paradigm [26]. Switch cue processing involved the activity of fronto-striatal regions, the superior parietal cortex, PCC, occipital cortex and precuneus. All these regions have been commonly identified as neural correlates of task-switching in previous studies [19,41–44]. Unlike these prior studies, in which attentional and response set shifting took place at the same time, the activity that we observed was associated with the presentation of the switch cue as compared with the repeat cue. Moreover, this was observed in a context in which switches took place after a series of trials, which allowed the stabilization of the task set, rather than in a context of rapid and frequent switches. Therefore, it can be associated with the reconfiguration of the stimulus-response associations that take place during cue processing [34]. Note, however, that we cannot fully dissociate the role of these regions during attentional (cue processing) and response (target) switching given that the cue-target interval remained constant throughout the fMRI paradigm (jittering between cues only). Likewise, the length (2 sec.) and fixed interval preceding the switch and non-switch target stimuli might explain the lack of association between brain activation during the presentation of the switching cue and the switch cost but, as suggested by Meiran [34], it is compatible with the notion of advanced reconfiguration.

### Reward Sensitivity and Cognitive Control

Individual differences in SR scores modulated the activation of the cortical and subcortical brain regions, which have been previously related to cognitive control during the presentation of switching cues. The medial prefrontal cortex, including different portions of the ACC, has been consistently reported to be involved in set switching [45–47]. The ACC negatively correlated with SR scores in both our current and previous switching studies [19], which strongly supports BAS modulation of brain activation in the ACC during task switching. This area is involved in the decision of invoking more or less executive resources a cost-benefit analysis of investing more or less attention to the task [7,48]. The lateral prefrontal cortex has been related to set switching and to other cognitive control processes, such as response inhibition or conflict detection [43,45,49]. Both the ACC and lateral prefrontal cortex have been proposed to be jointly involved in switching response rules and in preparing new action sets, presumably by updating information in working memory [50]. Likewise, other regions included in attentional networks, such as the superior parietal cortex and intraparietal sulcus [51,52], also showed a negative correlation with SR scores in the present study. These findings line up with the proposal of the ACC and lateral prefrontal cortex as key structures for the interaction between cognitive and motivational processes [4]. The negative association between the BAS and brain activity in a low-frequency switching task also coincides with findings from previous reports for working memory [20,35], which were interpreted as evidence of a higher neural efficiency in individuals with stronger BAS sensitivity. Our results are consistent with this interpretation. However, if we also consider the findings in our previous study [19], this association may not hold for the lateral prefrontal cortex with increased control demands. In other words, the need for increased control in a rapid task-switching context may be associated with more striatal and lateral prefrontal activity associated with the BAS. This would facilitate performance in such a paradigm [21], but this pattern may differ when switches are infrequent. Thus this area seems to interact with reward sensitivity as a function of task demands. More studies are required to delineate the relationship between reward sensitivity and lateral frontal activity, and to explore not only different cognitive control processes, but also other factors such as the presence of reward incentives, which seem to elicit increased activity in lateral prefrontal areas in high-SR participants [3].

In accordance with previous studies, which have indicated the involvement of the basal ganglia in task switching [41,53] and its relation to BAS sensitivity [19], we found that reward sensitivity modulated left ventral striatal activity during the processing of the switch cue. In our previous study [19], we found a positive correlation between SR scores and the right caudate during switch trials. In contrast, in the present study the left ventral striatum was correlated negatively with the SR scores. This discrepancy might be partly due to the strong response of the caudate in the current task, which is in line with the role of the striatum in cognitive flexibility [6]. Given that the ventral striatum has been positively associated with reward sensitivity in the presence of reward cues [18], absence of reward contingencies in the present task may have driven the opposite association. However, the present results do not support the association of BAS sensitivity with increased striatal activity as a mechanism for facilitation of cognitive flexibility. Instead, these findings indicate a generally reduced activity in the fronto-striatal regions involved in task switching in individuals with higher BAS sensitivity. A previous study also showed a similar pattern of reduced fronto-striatal activity in individuals with higher cognitive flexibility [54], which is consistent with the hypothesis of better task switching ability associated with the BAS. However, lack of BAS effects on behavior limits the interpretability of these findings.

We also found a positive correlation between SR scores and activity in the PCC, which was not initially hypothesized. Activity in this region has been reported in task switching [50,55,56]. In particular, the PCC function has been related to dopaminergic neurotransmission [57]. It has also been associated with the so-called default-mode network (DMN), which shows deactivation rather than activation in similar attentional tasks to that employed in the present study [58,59] and has been seen to predict performance in cognitive control tasks [60]. These results suggest the importance of the DMN in behavioral adaptation in goal-directed tasks and its suitable association with BAS sensitivity. In general terms, the present pattern of results shows that higher reward sensitivity is associated with reduced activity of the fronto-striatal brain regions involved in cognitive control. This provides evidence for a modulatory role of the BAS over cognitive processing and the activity of cognitive control brain areas, which may serve as a starting point for future studies to contribute to a better understanding of how this modulation operates.

Previous behavioral studies have indicated that individuals who are more sensitive to reward are faster at responding to switch trials when compared with individuals who are less sensitive to reward under high switching demands [21]. This has been interpreted as faster cognitive disengagement from previously relevant, and now irrelevant, stimulus attributes in high-SR scorers, which is in line with other cognitive studies of negative priming and latent inhibition [22,61] and would line up with the proposed association between appetitive motivation and cognitive flexibility [6]. However, other studies have shown that the BAS is associated with diminished activation in the ACC, lateral prefrontal cortex and parietal cortex during a working memory task, with no association with behavioral performance [20]. A similar pattern of altered brain activity without behavioral differences has been reported with clinical samples including ADHD and bipolar disorder patients, and substance-dependent individuals [62–67]. Lack of association between SR scores and switch costs in the present task, added to the correlation pattern found in the fMRI analysis, might be indicative of a differential recruitment of brain resources in goal-directed behavior, which nevertheless leads to comparable task performance levels in high- and low-SR scorers. As discussed before, two aspects are noteworthy: the present analyses were restricted to brain activity in response to a cue that did not require any immediate behavioral response, and the length and fixed character of the cue-target interval could have canceled the association between SR and subsequent switch-related behavior, similar to that observed between cue-related brain activation and the behavioral cost. Absence of BAS effects on behavior may limit the scope of our results to cue processing in task switching, which suggests that a pattern of attenuated response in frontal and striatal regions may underlie BAS modulation of switch-cue processing in the absence of reward contingencies. The fact that the results remained unchanged after controlling for the effect of the switch cost suggests that the activation pattern associated with reward sensitivity is independent of the behavioral effects of the task. We kept the foreperiod similar to the study of reference [26] and the switch cost effect remained significant in our study. However, our foreperiod can be considered long enough [34] to reduce the variability in performance that might have led to SR-related effects. In a future study, challenging foreperiods of shorter duration may clarify the influence of SR on prefrontal activation and the behavioral effects of cognitive set-switching.

### Summary and Conclusions

To summarize, the results of the present study indicate that BAS sensitivity is associated with reduced brain activity in the fronto-striatal regions involved in task switching and, generally, in executive function, which are proposed as candidate regions for the interaction between cognitive and motivational processes [4]. This is in accordance with the proposal of greater neural efficiency associated with the BAS under specific task demands [20]. However, our switch task did not include an experimentally induced motivational state (i.e., reward contingencies), which suggests that the interaction between cognition and motivational dispositions is important, even in purely cognitive tasks [19,21]. This interaction may help explain the adoption of different cognitive control strategies under diverse demanding task conditions.

These results also raise the question as to how this interaction between motivation and cognition occurs at the brain molecular level. Previous studies have highlighted the role of striatal and prefrontal dopamine in the interaction between motivational and cognitive control processes [68–71], and Gray [8] proposed the dopaminergic system to be the neural basis of the BAS. Therefore, future studies could explore the links among dopamine, individual differences and cognitive control via genetic imaging or pharmacological manipulations to dissociate the different processes involved in task switching.
